# Female perspectives of male partners’ inclusion in the prevention of mother-to-child HIV transmission programme in KwaZulu-Natal

**DOI:** 10.4102/curationis.v39i1.1691

**Published:** 2016-12-07

**Authors:** Mondli Miya, Tennyson Mgutshini

**Affiliations:** 1 Department of Nursing, University of Zululand, South Africa; 2 Faculty of Humanities, University of South Africa, South Africa

## Abstract

**Background:**

The South African government intervened by implementing the prevention of mother–to-child transmission programme (PMTCT) to curb the HIV transmission from mother to child during and after pregnancy. The PMTCT programme has been at the forefront of global prevention efforts since 1998. Without treatment, the risk of transmission ranges from one in five to one in two newborns; however, the risk of mother-to-child transmission can be reduced to as low as 2%–5% with evidenced interventions. Sub-Saharan Africa, and most particularly South Africa, is the most affected by the pandemic despite having the largest financial investment in PMTCT services across the continent.

**Objectives:**

The objectives of the study were to describe and explore the female perspectives of male inclusion in the prevention of mother-to-child HIV transmission programme in KwaZulu-Natal.

**Methodology:**

A qualitative, descriptive, explorative study was conducted through in-depth individual interview of pregnant women until data saturation.

**Results:**

The findings of the study revealed that the existing design of public hospitals was not wholly conducive to facilitating male inclusion in maternal and child health services. Resources were largely insufficient to support the participation of pregnant mothers and any attempts to support the inclusion of males needed to be based on a clear increase in service provision.

**Conclusion:**

The study recommended male partners’ inclusion in the prevention of mother-to-child HIV transmission to support effective management of HIV in pregnancy and PMTCT programmes. The inclusion of men will provide the holistic support needed by pregnant women on PMTCT programmes.

## Background

The Centre for Disease Control (CDC 2015) confirmed that over 34 million people globally were living with human immune virus and acquired immune deficiency syndrome (HIV/AIDS); 22.9 million (nearly 68%) of this represented infections within sub-Saharan Africa. In real terms, this translated into the fact that three out of every four people who were infected with HIVAIDS lived in sub-Saharan Africa. Affected by the HIV/AIDS epidemic, South Africa (SA), like other developing countries, has about 5.7 million people known to be living with the virus or disease (CDC 2015; World Health Organization (WHO) 2012a), which is the highest numbers of infected adults and children in the world. HIV/AIDS has had a noteworthy negative effect on life expectancy within Africa and, most specifically, within SA. On the continent of Africa, the average life expectancy has reduced from 59.4 years to 51.2 years primarily because of the impact of HIV and AIDS. However, this has since increased to 60 years in 2016 following several medical interventions such as anti-retroviral treatment (WHO 2012a, 2016). HIV/AIDS has proved particularly problematic because of a number of widely published unique characteristics, including the fact that (1) significant behavioural changes in the sexual practices of populations are required in order to manage the spread of HIV/AIDS, (2) the identity of a cure to the virus remains elusive, (3) HIV/AIDS has a wide symptom spectrum that is difficult to effectively manage and, finally, (4) the prognosis of affected individuals is correlated with the socio-economic status, that is, individuals from poorer backgrounds have much less favourable survival outcomes compared to their materially advantaged counterparts (WHO 2012b). This combination of factors places Africa at an increased risk of higher-than-average mortality rates when compared to other continents. This trajectory is borne out of statistics from the WHO which confirms the pandemic prevalence of HIV/AIDS.

HIV/AIDS represents the most significant clinical challenge facing health providers, clinicians and the world population. The high incidence of HIV/AIDS is reported in 33 countries globally, of which 22 countries are in the sub-Saharan region and the rest are distributed in Eastern Europe and Central Asia (UNAIDS 2011). Nationally, as of 2011, the estimated overall HIV prevalence was an estimated 5.38 million people (10.6% of the national population). Amongst those aged 15–49 years, an estimated 16.6% of the population are HIV positive (South Africa Statistics 2012). Annually, about 316 000 new infections are reported, with 63 000 (20%) of these new cases being reported in children between ages 0 and 14 years (Statistics South Africa 2012; WHO 2016).

The sub-Saharan region is not only the focal nexus of the HIV/AIDS burden but also the region where populations have the highest risk of continued infection (WHO 2010b). Of the 2.7 million newly infected adults and babies reported in 2010, 1.9 million (over 70%) were in sub-Saharan Africa (WHO 2011). This is at least eight times the rate of infection reported in South and South-East Asia, the region with the second highest rates of new cases at 270 000 (WHO 2011). Given the continued very high rates of infection, interventions to prevent transmission of HIV/AIDS have retained centre stage importance as the most promising means by which the impact of HIV/AIDS will be reduced with time.

The global HIV/AIDS reduction programme has identified six specific interventions that require prioritisation: (1) increasing the percentage of people who received a HIV test in the past 12 months and knowing their status; (2) increasing the proportion of people receiving anti-retroviral therapy (ART); (3) rates of reported condom use in last sexual encounter; (4) reduction of incidence, prevalence and death rates associated with tuberculosis and most relevant to the current study; and (6) the reduction in mother-to-child transmission of HIV (WHO 2012a). South Africa has adopted a new approach of managing HIV/AIDS, that is, the approach of testing and commencing treatment immediately irrespective of the viral load (WHO 2016).

HIV/AIDS is the leading cause of death in South Africa (WHO 2013). The epidemic disproportionately affects the young black and economically poor populations in South Africa (WHO 2010a, 2012a; 2013). Within South Africa, mother-to-child transmission represents a significant area of concern for two specific reasons. Firstly, the national rate of 2.8% of mother-to-baby transmission is appreciably above the target rate of 0% transmission by 2015 set by WHO. The uptake and continued adherence with prevention of mother–to-child transmission (PMTCT) health services is lower than expected and, most notably, KwaZulu-Natal province reports the highest provincial rate of mother-to-baby transmission (3.7%) in the country (KZN Health 2012; UNEFPA 2011). This is a particularly problematic pattern that adds to the need for specific research that considers the range of motivational factors and barriers related to PMTCT uptake.

The South African government has ensured that more than 2.6 million people are initiated on antiretroviral treatment by mid-2014 (Department of Health (DOH) 2015). South Africa has progressed well in HIV management of mother-to-child transmission. Ever since 2015, all South African women infected with HIV are commenced on lifelong antiretroviral treatment regardless of CD4 (lymphocytes) count or clinical staging (DOH 2015).

The Republic of South Africa (RSA), like all other developing countries, is affected by the HIV/AIDS epidemic, with about 3.5 million people known to be living with the virus or disease. HIV/AIDS is the leading cause of death in RSA (WHO 2010a) and this number has increased to 7 million people living with HIV, with 330 000 new infections reported annually in South Africa (WHO 2016).

### Problem statement

Since the initiation of PMTCT programmes in the year 2000 in SA, professional nurses have been at the forefront of PMTCT implementation (WHO 2007) and the PMTCT document used by professional nurses has been revised twice. At each review, there has been acknowledgement that the significant governmental investments have not been equalled by the reported success in PMTCT implementation and there is no evidence that men have been involved at any stage of PMTCT implementation in SA (KZN Health DOH 2012).

Many studies are largely focussed on issues related to the effective integration of PMTCT initiatives into mainstream services (Horwood *et al*. 2010), patients’ satisfaction, coverage and the available drugs utilised in PMTCT (KZN Health DOH 2012) neglecting the most significant support system stakeholder being the male. Notably, HIV/AIDS in pregnancy is not a female burden; instead, it should be shared by both parents. PMTCT services in South Africa are offered in almost all health facilities (98%). The percentage of HIV positive pregnant women receiving ART to reduce HIV transmission during pregnancy has steadily increased from 83% in 2009 to 87% in 2012 and mother-to-child transmission has decreased to 2.7% since 2011, and 0% HIV transmission from the mother to a child remains a challenge for all health workers and related stakeholders. Hence, this study sought to describe and explore the female perspectives of male inclusion in the prevention of mother-to-child HIV transmission programme at selected public hospitals in KwaZulu-Natal.

#### Research purpose

The purpose of the study was to explore and describe the female perspectives of male inclusion in the prevention of mother-to-child HIV transmission programme at the selected public hospitals in KwaZulu-Natal.

#### Research objectives

The objectives of the study were to:

- explore the barriers within the PMTCT programme to male inclusion
- describe the female perspectives of male inclusion in the prevention of mother-to-child HIV transmission programme at the selected public hospitals in KwaZulu-Natal.

## Research method and design

An explorative, descriptive, qualitative design was used. Creswell (2009) identifies qualitative research as being focused on an in-depth exploration of a phenomenon and can be presented in a narrative form, whose aim is to offer meaningful insights. In keeping with the principal tenets of qualitative research, as described by Creswell (2009), in-depth individual interviews were used to capture the depth and breadth of gender inclusiveness within an identified PMTCT programme.

### Qualitative research

A qualitative approach formed the basis of this research design. Qualitative research is person-catered and takes an ‘emic’ perspective (insider’s point of view). Thus, Creswell (2009) defines qualitative research as an enquiry process of understanding based on distinct methodological traditions of enquiry that explore a social or human problem in ways that support a researcher-built complex, holistic picture. This tradition analyses words, reports detailed views of informants and conducts the study in a natural setting. Therefore, the theoretical frameworks are not predetermined (Holloway & Wheeler 1996; Mouton & Marias 1990). A qualitative approach was used to explore and describe barriers towards male inclusiveness in PMTCT programmes.

For this study, the key qualitative aspects are related to the exploratory, descriptive and a contextually determined understanding. Each of these aspects is described below to emphasise the total study focus.

### Exploration

Adoption of an exploratory approach assisted the researcher to collect new data in areas where little or no research has been conducted (Mouton 1996; Mouton & Marais 1990; Woods & Cantazaro 1988). Cooper and Schindler (2001) state that an exploration typically begins with a search of published data. This was done in empirical exploration and included a description of barriers towards male inclusiveness in PMTCT programme. The researcher provided an in-depth description of experiences of HIV positive pregnant mothers regarding male inclusiveness in PMTCT programmes (Chinn & Kramer 1995; Mouton 1996). During conceptualisation of the findings, an accurate description of the relational statements formed the basis on which the recommendations were described.

### Description

Polit and Hungler (2004) state that descriptive research aims at giving insights into the characteristics of individuals, situation or groups and the frequency with which certain phenomena occur. During data collection (regarding the motivational factors and barriers towards gender inclusiveness in PMTCT), the researcher provided an in-depth description of the identified attributes towards male inclusiveness in PMTCT programme (Chinn & Kramer 1995; Mouton 1996). During the conceptualisation of findings, an accurate description of the relational statements formed the basis on which the recommendations were described.

### Contextualisation

According to Lincoln and Guba (1985), a phenomenon must be studied in its natural setting because individuals take their meaning from themselves within their context. Pregnant mothers who were directly involved with PMTCT programme in the selected hospitals were able to reveal particular information in a specific context (Babbie & Mouton 2011; Mouton 1996).

### Study setting

The study was undertaken in KwaZulu-Natal Department of Health which has six health districts. Within this, HIV positive pregnant women are referred to the larger hospitals for comprehensive HIV management in pregnancy. Three of these larger hospitals were selected in the KwaZulu-Natal province for the purpose of this study. The selected hospitals were as follows: one from Ethekwini Health District (192 patients attend the clinic daily), which is situated in the south of the KwaZulu-Natal; one from the central region of the province (164 patients attend the clinic daily), which falls under Mgungundlovu Health District; and one from Zululand Health District (210 patients attend the clinic daily), which is situated in the north of KwaZulu-Natal. The hospitals were selected to ensure geographical representation of the province. An additional criterion for inclusion was the large number of pregnant HIV positive patients managed by each of the mother-to-child transmission services.

### Population and target population

The study population consisted of patients on PMTCT programmes in three selected public hospitals in KwaZulu-Natal. Pregnant HIV positive patients were the primary target population for the study as the intended service users of PMTCT programmes.

#### Sample

Participants were purposively selected and were interviewed until data saturation. Data saturation happened upon the 13th interview. However, the researcher continued with two more interviews to confirm data saturation (*n* = 15). Thus, a total of 15 participants took part in the interview and saturation was confirmed.

#### Data collection

Data collection is the process of selecting and gathering data from participants. A researcher in qualitative interviewing has to take an open stance and consider all factors that determine choices of data collection, ranging from problem statement, research question and the aim of the research and clear description of the research methods in research design (Babbie 2010; Botma *et al.* 2010; Burns & Grove 2009). Data were collected by means of one-to-one interviews, making field notes and audiotaping.

Individual interviews involved communication between the interviewer and the pregnant women in the PMTCT programme, during which the interviewer sought to explore and describe the experiences of pregnant women regarding male inclusiveness in PMTCT programme.

Participants were interviewed individually for 45 minutes each in their hospital bed environment after being briefed about the study, and audiotaping and field notes were made simultaneously. During the interview, participants were encouraged to continue talking, using the necessary communication techniques such as probing. Participants were encouraged to elaborate on particular aspects of the discussion (Burns & Grove 2009).

The following research question guided the individual interviews:

Patients were asked: Please talk to me about your experience of involving your partner in this mother-to-child programme that you are part of.

#### Data analysis

Data were analysed by using the content analysis approach outlined by Elo and Kyngas (2008). Through this process, an inductive development of categories formed the basis of analysis. The aim was to build a post-analysis descriptive overview of the range of barriers and motivational factors for gender inclusiveness in the PMTCT programme. Within this, process standards were enacted to build a replicable and valid method for making specific inferences from the text (data collected) to other statements (data interpreted) or properties of its source (Mayring 2010).

Thematic and content analyses were used to analyse the data elicited from the exploration of the range of barriers and motivational factors for male inclusiveness within the PMTCT programme in public hospitals in KwaZulu-Natal as perceived by female patients on the same programme. Views expressed by participants were collated in general thematic groupings to generate ‘master-theme groupings’, and within each of these, more specific subthemes were identified. Finally, as a way of clarifying how commonly expressed themes were, the appearance of ‘descriptive terms’ was numerically counted and expressed through content analysis. Content analysis has been widely used in nursing studies as a systematic and objective method in quantifying phenomena (Creswell 2009). The researcher maintained accuracy in data analysis and interpretation by following all the steps of content analysis and categorising emerging findings.

#### Preparation phase

Firstly, three copies were made of the hand-written transcripts to facilitate notations within the transcription and the entire text was read. The research data were initially reviewed a number of times to ensure an in-depth understanding of the emerging themes within the text; the first reading was just a general overview of the text, while the second review focussed on identifying thematic words. Different colour pens were utilised to highlight the main themes and categories. These were then transcribed to a coding sheet. Content analysis facilitated exploring and describing the range of barriers and motivational factors for gender inclusiveness within the PMTCT programme in public hospitals in KwaZulu-Natal.

#### Open coding and establishment of categories

The second review focussed on underlining the thematic words, used to categorise the content into themes and subthemes. Within the text, ‘perception of roles’, motivating barriers in PMTCT programme as perceived by female patients, were all noted and specified as important emerging themes. Different colour pens were utilised to highlight themes and subthemes, which were then transcribed to a coding sheet. Content analysis was applied to facilitate a qualitative representation of emergent themes as they related to implementation processes used in the PMTCT programme. Participants’ perceptions of PMTCT male inclusiveness and their views about the way in which the programme was implemented were all documented.

## Ethical considerations

In ensuring full ethical adherence, the researcher approached the Department of Health in KwaZulu-Natal for initial discussion of the research project and to request permission to submit an application to utilise the chosen study sites ahead of obtaining academic ethical clearance from the Higher Degrees Committee in the Department of Health studies at University of South Africa In accordance with this articulated plan, a full proposal of the study was submitted and approved by the Department’s Higher Degrees Committee. A formal letter was submitted to the Department of Health requesting site permission for each of the study sites. Formal permission (ethics number, HSHDC/201/2013) to access sites was therefore granted. Explicit consent was sought from prospective participants 2 weeks before the initiation of the study and was later reconfirmed at the beginning of each of the interviews. The data collected were coded to ensure that there was no link to participants and only the researcher had access to the raw data and reassurances of commitment to confidentiality were made and reiterated throughout the study. The researcher respected and secured the well-being of the participants by refraining from any means of physical, emotional, spiritual or social discomfort and harm to participants, and ensured that participants had the right not to answer questions if they felt uncomfortable in answering and were allowed to withdraw from participating in the study without any repercussions, which is in line with the principle of non-maleficence described by Dhai and McQuoid-Mason (2011) as the method of avoiding harm in research conducting.

### Scientific integrity of the research

The researcher upheld the confidentiality of those involved in the interviews, keeping their anonymity and privacy secured; only the researcher had access to detailed information about the participants. The study was designed in such a way that risks of breaking confidentiality were minimised. The researcher has to ensure that the right to privacy and anonymity is upheld (Streubert & Carpenter 2011; Tolich 2008; Willig 2008). To minimise threats to internal validity, the researcher facilitated all the data collection. The researcher used the local language used by the community (Creswell 2012; Parahoo 2009; Polit & Beck 2012).

### Domain-specific ethical issues

In qualitative research, ethical conduct involves accountability to all different stakeholders and the unanticipated ethical concerns related to the ever-changing expectations of the study (Streubert & Carpenter 2011). In this study, the participants’ rights were observed in accordance with the international ethical principles as stipulated in the Belmont Report 1979 (Streubert & Carpenter 2011; Tolich 2008), the Medical Research Council Guidelines on Ethics for Medical Research and Guidelines for Good Practice in the Conduct of Clinical Trials in Human Participants in South Africa. Focus was on ensuring absolute protection and confidentiality of prospective participants, particularly as the subject matter related to HIV/AIDS is sensitive and can be a source of stigma for affected individuals. To minimise the risk of exposure, eventual participants had the option of selecting a venue of their choice at any of the available service delivery centres, and most participants opted for comfortable beds familiar to them.

## Results and discussion

The study was specifically focussed on eliciting the views of 15 HIV positive pregnant mothers, whose primary demographic characteristics (age, marital status and employment status) are explained in the next section.

### Biographical information

The biographical information is as follows: 15 interviews were conducted with the participants (*n* = 15), ages of the participants varied between 18 and 35 years of old, and the majority (87%) of participants were unemployed (*n* = 13).

### Pregnant mother interviews

The pregnant mothers (*n* = 15) took part in unstructured interviews. With each participant, a question was asked:

Please talk to me about your experience of involving your partner in the PMTCT that you are part of.

From this enquiry, participant responses related to the ‘structure’ of PMTCT services they had been involved with and from this a range of themes emerged. Participants felt that service policies dictated much of their experience and, in some instances, participants appeared unaware that males were allowed to be part of their antenatal care. In supporting the latter viewpoint, a quote from one of the participants is presented:

‘Wow! Government allows males to be with us while pregnant and delivering babies, wow! Very impressive, I will sell the idea to my partner.’ (Pregnant mother participant 2)

‘I was not aware that males are allowed to be part of PMTCT and even the department is written maternal services, so it never came to my attention that males are allowed [*sic*].’ (Pregnant mother participant 9)

From the above response, the suggestion was that the lack of awareness about an inclusive service structure was partially responsible for the non-engagement of male partners. The feeling that no other person would be allowed to support an expectant mother was expressed by others and included views such as:

‘I am not aware that our partners have a right to come with me.’ (Pregnant mother participant 5)

‘I was told children do not have to come with me to the clinic because I may be admitted at any time so I assumed same thing for my partner.’ (Pregnant mother participant 7)

One of the participants noted that the exclusion of a partner from her PMTCT sessions was not self-initiated but rather had been influenced by professionals, one of whom was quoted as saying:

‘All our maternal procedures here require females only, we walk around half naked, and I doubt if males can cope.’ (Quoted response from nurse professional as articulated by pregnant mother participant 8)

In addition to the participants identifying ‘service-structure’ as an important factor in the usage of PMTCT, the role ‘resources’ played in the experience of PMTCT services was specifically identified. Some of the expectant mothers noted that they were acutely aware that the issue of resources defined their own personal experiences of PMTCT services. The views were captured in some of the responses they offered, which included the following opinion:

‘At times the pharmacy runs short of ARVs and there are only two pharmacists that attend the entire hospital, so us as patients are exposed to long waiting periods for medication.’ (Pregnant mother participant 5)

‘A group of 5 nurses attend us, and we are more than 300 everyday; these nurses are human beings and they also get tired like everybody else.’ (Pregnant mother participant 11)

One of the participants spoke specifically about the possibility of engaging their male partners within their PMTCT and, within this, identified concerns about a general lack of resources as an important barrier. She indicated:

‘I am slightly concerned where extra nurses shall come from for such a bright idea of including males in PMTCT.’ (Pregnant mother participant 6)

The participant feedback about the impact that resources had on service delivery also highlighted the role ‘lack of space’ in clinics played as a likely barrier to the inclusion of men. Some participants felt that the limited space, the long waiting periods and concerns about privacy in the clinics made it difficult to allow males to participate and, for that reason, discussions about the involvement of males in PMTCT could only be contemplated if issues acknowledging the lack of space and long waiting periods in clinics could be satisfactorily addressed. Below are some of the expressed viewpoints that spoke to this observation:

‘Our clinic is too small for us pregnant women, so shall my man sit and wait for me here.’ (Pregnant mother participant 5)

‘Due to the forever increasing number of pregnant people with HIV, we end up waiting longer than expected.’ (Pregnant mother participant 9)

‘I see no need to bring my partner here, I have never seen any woman accompanied by partners.’ (Pregnant mother participant 1)

‘Women are half naked; my man will not find it suitable for him.’ (Pregnant mother participant 13)

‘Privacy is important to us, I have never seen a man toilet in this clinic, and I doubt if this clinic is ready for the gender inclusiveness.’ (Pregnant mother participant 7)

In addition to identifying limitations related to ‘lack of space’, and ‘privacy requirements’, factors related to personal circumstances were identified as having noteworthy influence on participants’ experiences of PMTCT. Lack of funds and the general unavailability of male partners because of work-related commitments were specified as potential barriers to the involvement of men. To support this, one of the participants indicated that:

‘My man has no money to travel monthly with me to the clinic, it is expensive. We both no working [*sic*] and we cannot afford transport fares.’ (Pregnant mother participant 3)

‘He is forever working during the weekdays and his employer cannot allow him to come here with me.’ (Pregnant mother participant 12)

There was widespread suggestion that the lack of financial resources had some negative impact on the possibility of widening PMTCT services to include males. Beyond that, participants also pointed to attitudinal factors that they felt were important considerations. For example, two of the participants expressed the following:

‘Nurses do not have patience for us, I am sure it shall be worse for our partners around since it shall mean extra work for them.’ (Pregnant mother participant 15)

‘Nurses do not seem friendly to people accompanying us, the complaining of huge numbers of patients turnover and I think they be more upset about our partners.’ (Pregnant mother participant 7)

‘…classes take long and my partner is working … suppose this could be time consuming for him …’ (Pregnant mother participant 3)

As part of the exploration, participants’ knowledge about key issues including their pre-existing knowledge about the PMTCT programme was elicited. The exploration focussed on participants’ knowledge of the drugs used in the PMTCT programme and to this a number of responses emerged in the discussion. These included:

‘Keep on changing on the same programme and I get confused.’ (Pregnant mother participant 11)

‘I was using three drugs initially and the nurse just changed and told me it the same thing, no enough information was given to me [*sic*].’ (Pregnant mother participant 6)

‘The knowledge given to us from our clinics to hospital does not match and we get confused on how often to return to the clinic [*sic*].’ (Pregnant mother participant 4)

### Limitation of the study

The study was conducted in three selected hospitals, and as such the sample was not wholly representative of their wider province of KwaZulu-Natal. Within that consideration, it is noted that the findings cannot be generalised to other areas in KwaZulu-Natal or South Africa. Although reassurances of confidentiality and anonymity were addressed in the information and consent sheet, it is possible that some participants offered responses that they thought would not impact future treatment – to this end, there was a specific acknowledged need for the research to evaluate whether participants’ answers reflected noteworthy levels of social desirability bias.

### Recommendations

The study offered a range of insights into participants’ perceptions as they related to the inclusion of males within their treatment programmes. To ensure application to wider practice contexts, participants’ views and the determinations from the background literature review were summarised into corrective service and practice recommendations as noted below.

*Service structure*: the design of service provision should be gender sensitive and make accommodations for males to be part of the serviced population. This may require additional monetary investment by health providers.

*Primary resource capacitation*: the current resources appear adequate to offer support to the women who attend MTCT services and, if any expansion in groups to be served occurs, then there will be a prerequisite need for a total review of resources to ensure that the inclusion of males does not further deprive expectant mothers from the scarce resources they currently have to share amongst themselves.

*De-stigmatisation interventions*: PMTCT services have inherent stigma-based challenges by virtue of their association with HIV/AIDS and this requires a concerted effort by service developers to work with local communities in educating them about HIV/AIDS to reduce the stigma associated with this area of health care.

## Conclusion

The results of this study highlight key barriers to the inclusion of males in PMTCT services and utilises these findings to propose specific recommendations to promote male inclusion. The engagement of service users, their partners and professionals simultaneously offers unique insights into this important area of study. Importantly, the study demonstrates that it is possible for researchers to simultaneously consider the attributions for male exclusion from the data in a way that has practical value to service users and service providers alike.
